# Elucidating the Anticancer Mechanisms of Tetrahydroxycurcumin: A Comprehensive Review of Preclinical Studies

**DOI:** 10.1002/fsn3.71125

**Published:** 2025-10-29

**Authors:** Muhammad Shahbaz, Ushna Momal, Hagar M. Mohamed, Asfa Perween, Hammad Naeem, Muhammad Imran, Muzzamal Hussain, Gamal A. Mohamed, Sabrin R. M. Ibrahim, Tadesse Fenta Yehuala, Suliman A. Alsagaby, Waleed Al Abdulmonem, Mohamed A. Abdelgawad, Ehab M. Mostafa, Samy Selim

**Affiliations:** ^1^ Department of Food Science and Technology Muhammad Nawaz Shareef University of Agriculture Multan Pakistan; ^2^ Department of Medical Laboratory Analysis, College of Medical & Health Sciences Liwa University Abu Dhabi UAE; ^3^ Department of Applied Medical Chemistry Medical Research Institute, Alexandria University Alexandria Egypt; ^4^ Department of Human Nutrition and Dietetics Muhammad Nawaz Shareef University of Agriculture Multan Pakistan; ^5^ Food Technology section, Post Harvest Research Centre Ayub Agricultural Research Institute Faisalabad Pakistan; ^6^ Department of Food Science and Technology University of Narowal Narowal Pakistan; ^7^ Department of Food Sciences Government College University Faisalabad Faisalabad Pakistan; ^8^ Department of Natural Products and Alternative Medicine, Faculty of Pharmacy King Abdulaziz University Jeddah Saudi Arabia; ^9^ Department of Chemistry, Preparatory Year Program Batterjee Medical College Jeddah Saudi Arabia; ^10^ Department of Pharmacognosy, Faculty of Pharmacy Assiut University Assiut Egypt; ^11^ Faculty of Chemical and Food Engineering Bahir Dar Institute of Technology, Bahir Dar University Bahir Dar City Ethiopia; ^12^ Department of Medical Laboratory Sciences, College of Applied Medical Sciences Majmaah University AL‐Majmaah Saudi Arabia; ^13^ Department of Pathology, College of Medicine Qassim University Buraidah Kingdom of Saudi Arabia; ^14^ Department of Pharmaceutical Chemistry, College of Pharmacy Jouf University Sakaka Saudi Arabia; ^15^ Department of Pharmacognosy, College of Pharmacy Jouf University Sakaka Saudi Arabia; ^16^ Pharmacognosy and Medicinal Plants Department, Faculty of Pharmacy (Boys) Al‐Azhar University Cairo Egypt; ^17^ Department of Clinical Laboratory Sciences, College of Applied Medical Sciences Jouf University Sakaka Saudi Arabia

**Keywords:** activator protein‐1, metalloproteinases, nuclear factor‐kappa B (NF‐κB), reactive oxygen species, tetrahydroxycurcumin

## Abstract

One significant reductive metabolite of curcumin, tetrahydroxycurcumin (THC), is a promising oncology candidate because of its multifunctional bioactivities. Preclinical data indicate that THC has a strong anti‐inflammatory, antioxidant, and anticancer profile, and as such, it is a better alternative to curcumin in treatment. Mechanistically, THC regulates important transcriptional factors that are involved in tumorigenesis, specifically nuclear factor‐kappa B (NF‐kB) and activator protein‐1 (AP‐1). Uncontrolled proliferation, inflammation, and resistance to apoptosis have been linked to aberrant activation of these pathways. Inhibition of NF‐kB and AP‐1 induced by THC suppresses cancer cell survival signaling and triggers apoptotic cell death. Simultaneously, THC inhibits the action of matrix metalloproteinases (MMPs), which are involved in the degradation of extra cells and metastatic spread, and promote tumor invasion and metastasis. Experimental research has also shown the effectiveness of THC in various cancers such as breast cancer, prostate cancer, colon cancer, and skin cancer. It is important to note that THC increases the therapeutic index of traditional chemotherapeutics, where they show synergistic interactions and counteract drug resistance mechanisms, which is a key obstacle in clinical oncology. THC has better physiological stability and bioavailability compared to its parent molecule, a characteristic that alleviates one of the greatest translational limitations of curcumin. This review highlights the molecular processes underlying the anticancer action of THC, its possible use as a single agent and as an adjuvant to already established chemotherapeutic protocols, and the translational issues that need to be overcome to achieve clinical acceptance. Together, the existing evidence supports THC as an attractive future cancer therapeutic with the potential to improve treatment outcomes and overcome drug resistance.

## Graphical abstract

Tetrahydroxycurcumin is an attractive multifunctional compound in cancer‐driven therapeutics. Its anticancer potential in modulating a wide range of oncogenic signaling networks, combined with its dual properties as an antioxidant and anti‐inflammatory agent, highlights its potential to become a transformative anticancer drug. One of the principal curcumin metabolites that has gained significant interest in oncological studies is tetrahydroxycurcumin (THC) due to its strong anticancer, antioxidant, and anti‐inflammatory effects. It has been shown that THC has a regulatory effect on several key signaling pathways related to cancer control, such as nuclear factor kappa B (NF‐kB), activator protein‐1 (AP‐1), and matrix metalloproteinases (MMPs). By regulating such pathways, THC stimulates apoptosis, tumor suppressor functions, and inhibits the spread of metastasis.

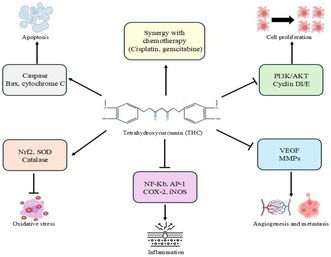

## 1 Introduction

Numerous chronic disorders, such as neurological, metabolic, and cardiovascular syndromes, are being studied in detail in relation to free radicals. “Reactive oxygen species” (ROS) are oxygen‐centered intracellular species, such as hydrogen peroxide, superoxide radical, and hydroxyl radical. Enzyme metabolism (including amine oxidase, xanthine oxidase, and other similar enzymes), mitochondrial electron transport chains, or responses to environmental stimuli (such as UV rays, chemicals, or pathogen attacks) are the processes that produce these species. The cellular enzymatic machinery or non‐enzymatic compounds are then removed or eliminated (Pospíšil et al. 2022). According to the free radical theory of aging, cellular aging may be brought on by oxidative stress, a condition in which ROS generation exceeds ROS scavenging and DNA repair processes. Serum C‐reactive protein and urine F2‐isoprostane, two indicators of oxidative stress and inflammation, respectively, sharply decreased in 285 adolescents who consumed more fruits and vegetables (rich in vitamin C and polyphenols). Citrus flavonoids have anti‐inflammatory and ROS‐scavenging qualities that show neuroprotective benefits in vitro (Evans et al. 2022).

**FIGURE 1:**
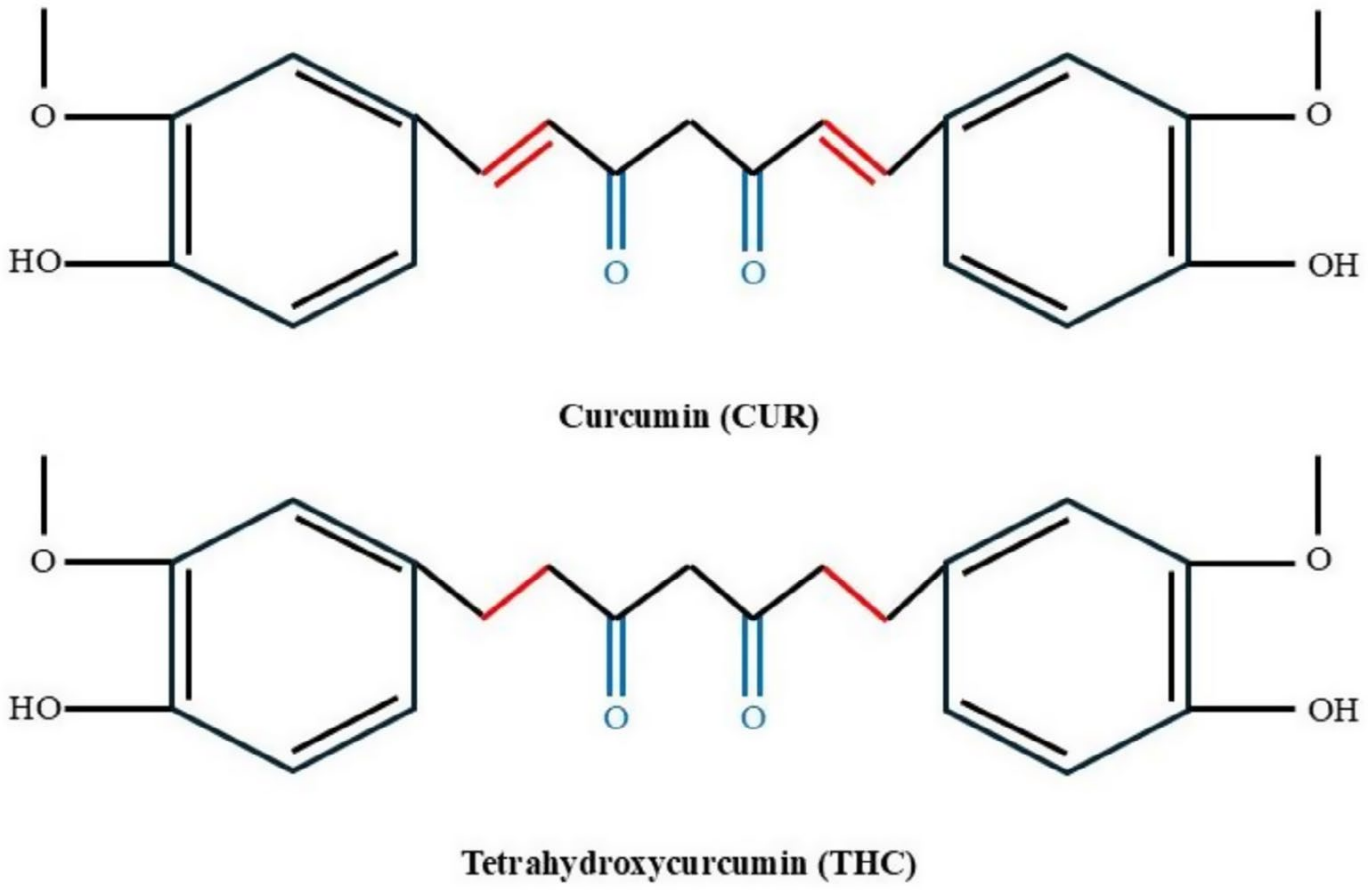
Structure of curcumin and tetrahydroxycurcumin.

The antioxidant and radical‐scavenging properties of natural compounds such as yam dioscorin, curcuminoids, tannin hydrolizable geraniin, and synthetic peptides of in silico pepsin hydrolysis are thus covered in many studies. The Maillard reaction starts with the non‐enzymatic glycation of carbonyl compounds in proteins to reduce sugars and nitrogen groups through nucleophilic attack, producing Shif Base and Amadori products. The reaction's final step produces irreversible advanced glycation end‐products (AGEs) (Ruan et al. 2018). Additional structurally active byproducts of sugars and fatty acids are the AGEs, N eta‐(carboxymethyl) lysine, and the metabolites glyoxal and methylglyoxal. By acting on the AGE receptors, the AGEs can increase ROS generation through NADPH oxidase. The rate of reaction of d‐galactose in non‐enzymatic glycations is roughly 4.7 times that of d‐glucose when hemoglobin is utilized as a targeted protein in vitro. As a result, in a rodent model, prolonged galactose administration frequently causes oxidative stress, and the components of the hippocampus that depend on spatial memory are gradually deteriorating. It has been observed that antioxidant therapies improve memory and spatial learning deficits. Turmeric, a spice used in food and medicine, is derived from the rhizome of *Curcuma longa* . In turmeric formulations, curcumin, tetrahydroxycurcumin, demethoxycurcumin (DMC), and bisdemethoxycurcumin (BDMC) are all referred to as “curcuminoids” [1,7‐bis(3,4‐dihydroxyphenyl)‐1,6‐heptadiene‐3,5‐dione]. Demethylcurcumin [1‐(3,4‐dihydroxyphenyl)‐7‐(4‐hydroxy‐3‐methoxyphenyl)‐1,6‐heptadiene‐3,5‐dione] and demethyldemethoxycurcumin (demethyl‐DMC) [1‐(3,4‐dihydroxyphenyl)‐7‐(4‐hydroxyphenyl)‐1,6‐heptadiene‐3,5‐dione] have also been detected in trace amounts in curcuminoids (Sharma and Sharma 2022). Figure 1 represents the structural difference between curcumin (CUR) and its major metabolite, tetrahydroxycurcumin (THC). The similarity between the two compounds is that their backbone is made up of two aromatic rings, which are substituted with hydroxyl and methoxy groups, having two keto groups on the linker between them. The central chain difference is that curcumin has two conjugated double bonds (highlighted in red) that are a component of its α, unsaturated diketone system, which makes it chemically reactive and unstable. Conversely, the reduction of these double bonds makes tetrahydroxycurcumin, which does not have a conjugation reaction and is more stable as a saturated diketone. This topographical alteration increases the chemical stability and bioavailability of THC over curcumin without affecting its antioxidant and therapeutic activity. As a result, THC has frequently been considered a more medically promising derivative to be used in biomedicine, most specifically in cancer treatment (Liu et al. 2019).

In contrast to their primary constituents, curcumin, DMC, BDMC, and THC, which have been documented to have antioxidant effects in vitro, minor fractions of curcuminoids have little recognized biological activity. It was recently observed that pretreatments with demethylcurcumin and demethyl‐DMC, rather than curcumin, DMC, and BDMC at similar concentrations, decreased the cytotoxicity of HaCaT keratinocytes treated with hydrogen peroxide. Compared to the more prevalent turmeric compounds, such as curcumin, demethoxycurcumin, and bisdemethoxycurcumin, researchers have suggested that THC has more stability within the pH range since it is a reductive metabolite. When compared to other pharmacologically inert curcumin metabolites, such as curcumin glucuronide and curcumin sulfate, tetrahydroxycurcumin (THC) retains a very strong pharmacological activity, especially in its remarkable antioxidative qualities.

In comparison to THC, curcumin also contains curcumin glucuronide, DHC‐glucuronide, octahydrocurcumin (OHC), hexahydrocurcumin (HHC), dihydrocurcumin (DHC), and curcumin sulfate. Furthermore, it has been demonstrated that a number of these metabolites possess anti‐inflammatory and antioxidative qualities (Sobhani et al. 2021). In a study where it inhibited the formation of COX‐2, which was shown to be low in contrast to curcumin, THC demonstrated anti‐cancer characteristics and antioxidant actions on mouse macrophages. By producing a significant accumulation of SW480 cells in the G1/G0 phase of the cell cycle, THC has shown that it can induce cytotoxicity and work in conjunction with 5‐fluorouracil to stop the growth of HT‐29 colorectal cancer cells (Câmara et al. 2022).

Additionally, THC possesses antioxidant and anti‐inflammatory qualities. Compared to curcumin, it dramatically reduced the amount of NF‐κB activity inhibition. Nuclear factor‐kappa B (NF‐kB) is a family of inducible protein complexes of transcription factors that is central to the regulation of immune response, inflammation, cell proliferation, apoptosis, and survival functions that are vital in cancer, inflammatory diseases, and autoimmunity (Guo et al. 2024).

In contrast to curcumin, it exhibits comparatively substantial DPPH scavenging action and antioxidant potential, preventing AAPH‐induced oxidation of linoleic acid. Another metabolite of curcumin, curcumin sulfate, is physiologically active when compared to curcumin, but with less activity, specifically in inhibiting PGE2 activity (Pandey et al. 2020).

## 2 Bioavailability of Tetrahydroxycurcumin

Curcumin is converted to curcumin glucuronide and curcumin sulfate via metabolic O‐conjugation, and it is bioreduced in animals to provide THC, hexahydrocurcumin, and hexahydrocurcuminol. Consequently, curcumin's oral bioavailability in vivo has been incredibly low. However, the active metabolic component THC in tissue and plasma explains curcumin's bioavailability. One study found that rats given Curcuma‐P for 4 weeks had subcutaneous adipose tissue containing THC (235 ± 78 ng/100 mg tissue), but not curcumin. THC and curcumin, however, were not found in the mouse plasma. Rats' liver and stomach cytosols have been shown to contain THC. It is regarded as the form of curcumin that is accessible in vivo due to its stability in plasma and greater resistance to curcumin in physiologic buffer solution (pH 7.2). THC appeared in mouse plasma treated with curcumin after 80 min, but curcumin was not identified. However, curcumin was found in the brains of mice that received curcumin treatment. A published study of mice administered curcumin revealed the same effect (Ipar et al. 2019). Along with these, it is shown that THC is more stable than curcumin. THC and curcumin had respective half‐lives of 813 and 186 min for degradation in cell culture media. They each had half‐lives of 111 and 232 min in plasma. Additionally, THC and curcumin have been shown to more than double the absorption of epigallocatechin‐3‐gallate in HT‐29 and MDCKII/MRP1 cells (Bolger et al. 2018). However, a number of investigations into the bioavailability of curcumin have discovered that even a small amount of curcumin is accessible in animal serum. For example, rats who received 2 g/kg of curcumin orally showed a blood concentration of 1.35 ± 0.23 μg/mL at 0.83 h; whereas, individuals receiving this dosage had either very little or no detectable curcumin in their serum (Tavčar and Vidak 2024). An oral dose of 3.6 g curcumin was shown to have a plasma curcumin content of 11.1 nmol/L 1 h after the dosing in a different investigation conducted in a human clinical trial. When tetrahydroxycurcumin was administered intravenously, as opposed to orally, there was adequate availability of curcumin in the plasma. After being injected into the blood plasma at a dose of 2 mg/kg through the tail vein, it was at 6.6 μg/mL (Hegde et al. 2023).

## 3 Antioxidant Potential

Tetrahydroxycurcumin (THC) is a curcumin‐related metabolite that has received considerable attention due to its antioxidant potential. Antioxidants remove reactive oxygen species (free radicals), which are necessary to cause oxidative stress with aging and other chronic diseases, including cancer. Similar to curcumin, THC is also an effective scavenger of free radicals, but unlike the latter, its ability to perform this task is not restricted by low bioavailability and stability, which further makes it a superior choice to be implemented in therapy targeting the treatment of oxidative stress diseases (Chen et al. 2021). Hence, tetrahydroxycurcumin has been found to have great antioxidant capacity via a variety of activities, which include inhibition of lipid peroxidation and eradication of free radicals, including hydroxyl, superoxide, and DPPH (1,1‐diphenyl‐2‐picrylhydrazyl). Studies on oxidative stress have treated cannabidiol as a cell‐preservative through the up‐regulation of antioxidants, catalase, and superoxide dismutase (SOD). The enzymes assist in preventing the damage that oxidative stress inflicts on the cells by clearing these ROS from the cell (Kao et al. 2020). This aids in mitigating the occurrence of the related disease. Also, it has been shown that tetrahydroxycurcumin enhances the activity of the Nrf2 (nuclear factor erythroid 2‐related factor 2) pathway, which is paramount in antioxidant protection of our bodies. Activation of this pathway stimulates the genes for the production of antioxidant enzymes, another example of the description of THC as a cell protector against oxidative stress (Zheng and McClements 2020).

**FIGURE 2:**
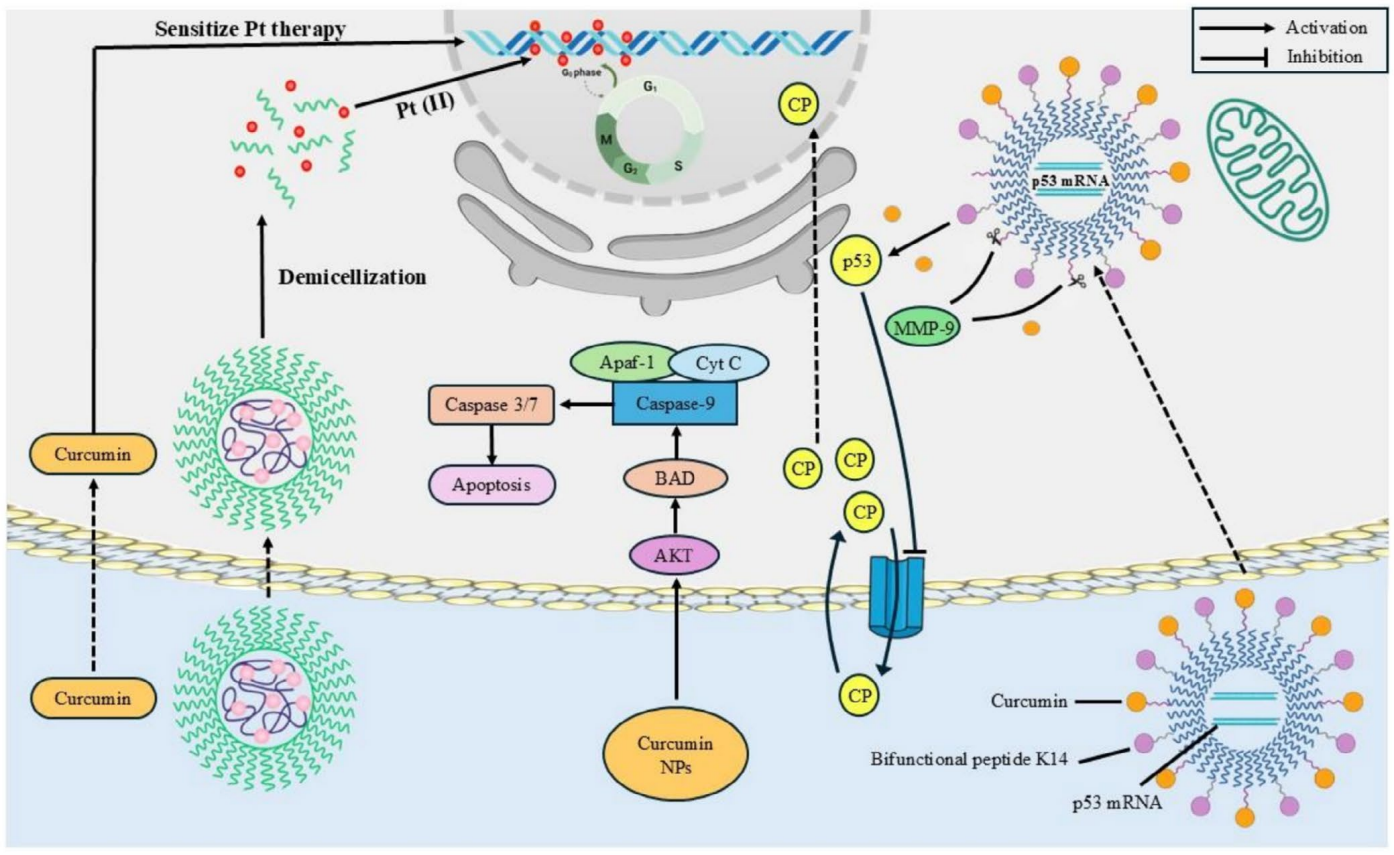
Nanoparticles for curcumin in cancer chemotherapy.

Tetrahydroxycurcumin has potential beneficial applications in treating other disorders such as diabetes, cancer, neurodegenerative, and cardiovascular diseases, as oxidative stress has been implicated in the occurrence of these disorders. Some of the compounds that may be developed in the future include tetrahydroxycurcumin, which has the potential to target the molecular level of oxidative damage. The peroxidation of erythrocyte membrane ghosts indicated antioxidant activity that also demonstrated a more potent activity in THC than in curcumin. They also demonstrated that the β‐diketone derivative of THC must be able to exhibit antioxidative capacity through the cleavage of the active methylene carbon C‐C of the two carbonyl compounds. The ability of the free radical scavenging was measured using the DPPH scavenging assay, which displayed that the THC steroid was superior to curcumin (Saini et al. 2023). Figure 2 demonstrates that curcumin, the parent compound of tetrahydroxycurcumin, and particularly curcumin nanoparticle, improves chemotherapy sensitivity, induces mitochondrial apoptosis, blocks pro‐survival AKT signaling, and recovers p53 pharmacologic activity to prevent tumor growth and metastasis (Kao et al. 2020).

## 4 Anticancer Perspectives

Tetrahydroxycurcumin (THC) is a metabolite of curcumin that has shown strong anticancer properties through multiple chemical effects. Research has revealed that tetrahydroxycurcumin possesses various anti‐cancer properties, which include the ability to modulate several signal pathways associated with the development of cancerous cases. Such pathways are angiogenesis, metastasis, cell cycle control, and apoptosis (Lai et al. 2020). The primary mechanism of action of tetrahydroxycurcumin against cancer is through modification of the transcription factors, including inflammatory diseases and cell survival tools, including nuclear factor‐kappa B (NF‐κB) and activator protein‐1 (AP‐1). Such pathways are often dysregulated in the cancer cells, and that has a role in making the tumors resistant to therapy. Tetrahydroxycurcumin has demonstrated its capacity to suppress apoptosis and cancer cell growth in many cancer models through modification of these processes. Moreover, the interaction between tetrahydroxycurcumin and the metastases of the cancer cells has also been widely researched. It prevents the work of matrix metalloproteinases (MMPs), body substances that promote invasion and proliferation of cancerous cells to the adjacent organs. The results render tetrahydroxycurcumin a prospective metastasis prophylactic, which happens to be a primary challenge of cancer treatment (Singh et al. 2022). Regarding the improvement of the effectiveness of traditional cancer treatment methods, tetrahydroxycurcumin has been shown to enhance chemotherapy and lower the side effects. During the process, it is known to augment anticancer effects of cisplatin by inhibiting drug resistance mechanisms as well as enhancing the overall effectiveness of the therapy at a cellular level (Cheng et al. 2021).

1,2‐dimethylhydrazine dihydrochloride, a potential carcinogen of the colon in animals, resulted in colon carcinogenesis that was used as an end‐point marker lesion by treating animal models of colon carcinogenesis. These models demonstrate that the active level of THC was more effective than curcumin in preventing the formation of aberrant crypt foci and cell proliferation in mouse skin carcinogenesis triggered by 7,12‐dimethylbenz[a]anthracene. However, it was less effective than curcumin in preventing the induction of TPA‐induced ornithine decarboxylase activity and carcinogenesis (Moulahoum et al. 2018). A different study found that curcumin was less efficient than THC at preventing the colon carcinogenesis caused by azoxymethane. The reduction of iNOS and COX‐2 levels in the colon tissue of the mice treated with AOM has also been found to be mediated by the inhibition of ERK1/2 activation, Wnt‐1, and ‐catenin protein production, and GSK‐3beta phosphorylation. There was also a decrease in the level of the protein of connexin‐43, which is a significant molecule of the gap junctions (Sorgen et al. 2018). Also, experiments have shown that tetrahydroxycurcumin exhibits high binding affinity to a wide range of target proteins, stabilizing protein‐ligand complexes that are suspected and paramount in the process of cell survival and apoptosis regulation. Various structural modifications on curcumin have been done in silico, which have found tetrahydroxycurcumin as a potential strong binding protein component of cancer progression as a drug agent. Tetrahydroxycurcumin can be offered as an effective treatment agent alone or in combination with other chemotherapies. It can affect multiple molecular pathways and inhibit cancer cell growth, which makes it a perfect object of additional clinical studies (Amaroli et al. 2024). The key anticancer mechanisms of THC across different cancer types are summarized in Table 1.

**TABLE 1:** Anticancer mechanisms of tetrahydroxycurcumin (THC).

Cancer type	Mechanism of action	Key molecular targets/pathways	References
Breast cancer	Suppresses proliferation and invasion, promotes apoptosis, cell cycle arrest and modulates miRNA expression	NF‐ΚB inhibition, PI3K/Akt blockade, cyclin D1/CDK4 inhibition	Wang et al. (2019)
Gastric cancer	Induce apoptosis, inhibit tumor growth, suppress lymphangiogenesis	Suppress Prox‐1, VEGFR, LYVE‐1, inhibit NF‐ΚB pathway	Koh et al. (2021)
Leukemia	Induce apoptosis, enhance imatinib efficacy	Activates caspase‐3, caspase‐8 and caspase‐9, down regulate NF‐ΚB subunits, p210, p50/p65	Mutlu Altundağ et al. (2021)
Skin cancer	Induce apoptosis, prevent UV‐induced oxidative damage	Caspase activation, downregulates COX‐2/iNOS, ROS scavenging	Trivedi et al. (2017)
Liver cancer	Induces apoptosis, downregulates angiogenesis and inhibits proliferation	Inhibition of Notch 1 signaling, suppresses VEGF‐A, reduces COX‐2	Hu et al. (2016)
Colon cancer	Induces apoptosis, anti‐inflammatory effect and inhibits cell growth	Downregulates COX‐2, iNOS, suppresses NF‐ΚB, inhibits MMP	Malki et al. (2020)
Ovarian cancer	Induces apoptosis, suppresses autophagy and reduces cisplatin resistance	Inhibits SERCA, activates Nrf2/HO‐1, suppress AKT/mTOR, inhibits STAT3/NF‐ΚB	He et al. (2016)

### 4.1 Breast Cancer

Breast cancer affects the tissue of the mammary glands, and the epithelial cells lose their ability to regulate their cell proliferation. The ducts or lobules can initiate tumor formation, which is called in situ cancer. It can subsequently spread by the lymphatic and the blood system to the lymph nodes and systemic tissues. Old age, obesity, lack of physical activity, family history or mutations in genes (BRCA1, BRCA2, TP53, PTEN, PALB2, STK11, NF1), exposure to external hormones or radiation, and alcohol and tobacco use are among the numerous risk factors linked to an increased risk of breast cancer (Ajabnoor 2023). Tetrahydroxycurcumin is also anti‐cancerous, particularly with breast cancer. Studies have shown that tetrahydroxycurcumin contains several biological processes that work together to reduce the size of the tumor, making it a great multifaceted treatment for breast cancer. A layer at the core of Wnt is the β‐catenin protein (Vallée 2022). Tetrahydroxycurcumin also promotes cell degeneration, reduces the PI3K/Akt pathway, and prevents angiogenesis, growth, and cell proliferation. By making the malignant breast cells die, this technique affects their autophagy and apoptosis. Additionally, tetrahydroxycurcumin prevents the tumor and cell proliferation‐promoting epidermal growth factor receptor (EGFR) pathway from being activated. Due to the decrease in the number of EGFR proteins on the cell surface, tetrahydroxycurcumin inhibits the growth of tumor cells and makes their proteins insensitive to their ligands (Pengjam et al. 2021). NF‐kappa B is one of the primary transcription factors involved in the proliferation of breast cancer cells, which become activated when exposed to certain cytokines, TNF‐alpha, and interleukins, which are both necessary in the transcription process of an oncogene.

Tetrahydroxycurcumin prevents the phosphorylation of NF‐ kB and hence arrests the activity of these transcription functions and halts the proliferative pathways (da Silva Lopes et al. 2024). Moreover, curcumin increases ROS production and also induces the crosstalk of breast cancer apoptosis, Bax‐mediated. Tetrahydroxycurcumin governs cell invasion, migration, and proliferation quite differently, depending on the type of cell. It is a regulator of the network of glass RNA and microRNA interaction in the breast cell. Tetrahydroxycurcumin can also reduce the production of tumors and promote the reduction of miR‐19a and miR‐19b in BLBC cells, as well as increase the miR‐181b levels, miR‐34a, miR‐16, miR‐15a, and miR‐146b‐5p levels. The ability of miRNA expression regulation makes an enormous contribution towards the prevention of the development of tumors, as well as to apoptosis (Wang et al. 2019). Tetrahydroxycurcumin inhibits the activity of cyclin/CDK4 and cell division by blocking the interaction between Cyclin D1 and Cyclin‐dependent kinase 4 (CDK4). Cyclin‐dependent kinases (CDK) inhibitors are also suppressed in tandem with the induction of natural degradation of the cyclin E that is overexpressed in breast cancer. The results of these activities are inhibitory to the G1 phase of cell division. Moreover, tetrahydroxycurcumin induced DNA degradation and apoptosis of breast cancer cells. The antiproliferative, antimigratory, and antimetastatic effects of tetrahydroxycurcumin in TNBC were mediated through regulation of the Hedgehog (Hh)/Gli1 signaling cascade and up‐regulation of Glioma‐associated oncogene homolog‐1 (Gli1). The occurrence of these processes indicates the characteristic of the tetrahydroxycurcumin compound as a potential therapeutic agent in influencing tumor progression and the significance of the compound in the development of anti‐cancer treatment strategies (Zhang, He, et al. 2020). These include directing therapeutics to tumors, where high concentrations of therapeutics can be achieved at the tumor site and result in a strong anti‐tumor effect, making this a field of active interest. Most local treatments require adequate exposure of tumors, which may be based on the pharmacokinetics of the agent. The anticancer activity of Au‐polyvinylpyrrolidone nanoparticles loaded with folate‐curcumin (FA‐CurAu‐PVP NPs) was applied intratumorally to the 4 T1 tumor in 6‐week‐old BALB/c mice. This increased the discharge of tetrahydroxycurcumin 8‐fold in this nano‐formulation, as it was able to concentrate in the tumor through folate receptors and its progressive discharge of the drug within the tumor. Similar results were recorded in mouse models, which contain TNBC cells (BT‐549 cells), with reduced tumor growth in mouse models intratumorally receiving tetrahydroxycurcumin‐loaded phosphorylated calixarene micelles (Mahalunkar et al. 2019). The effects of injecting mice with surgically excised tumors (4T1 cells) with thiolated chitosan‐coated liposomal hydrogel (CSSH/Cur‐Lip gel), a curcumin delivery method, were investigated. In situ tumor recurrence rate, toxicity, and survival duration were all significantly reduced by CSSH/Cur‐Lip gel. The gel provides an extracellular environment that delays the distribution of tetrahydroxycurcumin and hinders tumor reoccurrence (Li et al. 2020).

A 52‐year‐old female patient who had stage II triple‐negative breast cancer reported a partial response to the conventional combination of doxorubicin‐cyclophosphamide therapy but progressive resistance. An experimental adjunct therapy was initiated based on preclinical evidence of better solubility, bioavailability, and anticancer action of tetrahydroxycurcumin (THC) than curcumin. The patient was given an oral formulation of THC as well as chemotherapy. Within 12 weeks, tumor markers (such as Ki‐67) and NF‐kB activities were reduced, with an increase in apoptotic indices, which is in line with the mechanistic role of THC in redox‐sensitive transcription factor regulation and suppressing matrix metalloproteinases. There was stabilization of tumor progression clinically and improvement of chemosensitivity with minimal extra toxicity. This preclinical case highlights the translational potential of THC as a synergizing adjuvant in resistant breast cancer, which is consistent with preclinical evidence that the compound can improve chemotherapeutic activity and overcome resistance (Lai et al. 2020).

### 4.2 Pancreatic Cancer

Pancreatic ductal adenocarcinomas (PDACs), which account for over 90% of all exocrine pancreatic cancers, have a poor prognosis and are among the most common causes of cancer‐related fatalities. Due to the high rate of recurrence and the inability to resist the medication, patients are usually discovered at a late stage, which limits the alternatives for therapy. Therefore, to improve patients' quality of life or chances of survival, new therapeutic approaches that could improve the currently accessible and successful therapy or that are more valuable are required. A naturally occurring substance called curcumin is taken from *Curcuma longa* . The medicinal potential of curcumin and related curcuminoids, including tetrahydroxycurcumin, has been the main focus of research in recent decades because of its low toxicity and pharmacological safety. Numerous biological actions of curcumin have been found through research. Its pharmacological characteristics include anti‐inflammatory, anti‐angiogenesis, antioxidant, and anticancer potential, among others. The anti‐proliferative and pro‐apoptotic characteristics of curcumin are responsible for its ability to inhibit tumor growth. These effects are mediated through a cascade of molecular pathways such as NF‐kB, JNK, and ERK pathways, among others. Given the fact that EGFR‐related pathways are also considered to be pathogenic in PDAC, curcumin can act against PDAC by interrupting the pathways (Wang et al. 2021). Based on this, it has been claimed that deregulation of the EGFR pathway is an important aspect in the pathophysiology of PDAC. The overexpression of EGFR in PDAC seems to be a prognostic indicator in terms of patients. Tetrahydroxycurcumin has been experimented on pancreatic cancer cells in three different cancer cell lineages, Panc‐1, BXPC‐3, and L3.6pl. Such results have shown that curcumin can cause apoptosis through the suppression of the NF‐κB pathway and the downregulation of EGFR.

Pancreatic ductal adenocarcinomas (PDACs), which account for more than 90% of all exocrine pancreatic malignancies, have a very poor prognosis and are a major cause of cancer‐related mortality. The majority of patients are diagnosed at an advanced stage, and treatment choices are restricted due to the prevalence of relapse and drug resistance. Therefore, some novel therapeutic measures are required to enhance the patients' quality of life or survival, which may be more beneficial or superior to the existing, successful treatment. Curcumin is naturally found in the rhizome of *Curcuma longa* . The medicinal potential of curcumin and related curcuminoids, particularly tetrahydroxycurcumin, has been the subject of decades' worth of research due to its low toxicity and pharmacological safety. It has been determined that curcumin exhibits several biological effects. Among other pharmacological actions, anti‐inflammatory, anti‐angiogenesis, anti‐oxidant, and anticancer properties have been documented. Curcumin's ability to eliminate tumors is attributed to its anti‐proliferative and pro‐apoptotic properties. One of the biochemical pathways through which the actions are performed is the suppression of the NF‐κB, JNK, and ERK signaling. The second mechanism is that curcumin can treat PDAC by interfering with its pathogenic pathways, affecting EGFR (Wang et al. 2021). It was stated that the deregulation of the EGFR pathway is a primary cause of PDAC development. EGFR overexpression is one of the potential prognostic factors in PDAC patients. Several cancer cell lines, including Panc‐1, BXPC‐3, and L3, have been treated with tetrahydroxycurcumin. According to these findings, curcumin may cause apoptosis by downregulating EGFR and then inhibiting the NF‐κB pathway to cause apoptosis. In a different study, tetrahydroxycurcumin decreased cell survival and promoted apoptosis in lung and pancreatic cancer cell lines by downregulating EGFR and cyclooxygenase‐2 (COX‐2) (Liu et al. 2024).

Also, another study has revealed that curcumin inhibits pancreatic cancer cell growth; this is applicable at different times and dose levels, but is through the inhibition of the NF‐CAP pathway. In one study, curcumin was proven to have an anticancer effect on Panc‐1. After curcumin treatment in BXPC‐3 human pancreatic cancer cells, cell cycle arrest was observed at the G2/M phase, which also induced apoptosis through ATM/Chk1. Scientists have examined the influence of curcumin modulation on the expression of various cell lines of pancreatic cancer. These results indicated that this drug will induce pancreatic cell lines' apoptosis and growth arrest. Tolfenamic acid and tetrahydroxycurcumin combination displayed a stronger anti‐proliferative response on Pc cells L3.6pl and MIA Paca‐2 since they inhibited Sp1 and induced NF‐κB nucleus to translocate (Munir 2021). Besides, curcumin has been described as anticancer against various forms of cancer. In particular, two cancerous cell lines, SGC‐7901 and BGC‐823, were selected to investigate the effects of tetrahydroxycurcumin on cell proliferation in pancreatic cancer cells. They found that tetrahydroxycurcumin was capable of up‐regulating miR‐33b in pancreatic cancer cell lines, which was found to cause cell death and reduce cell proliferation (Morshedi et al. 2021).

### 4.3 Prostate Cancer

**FIGURE 3:**
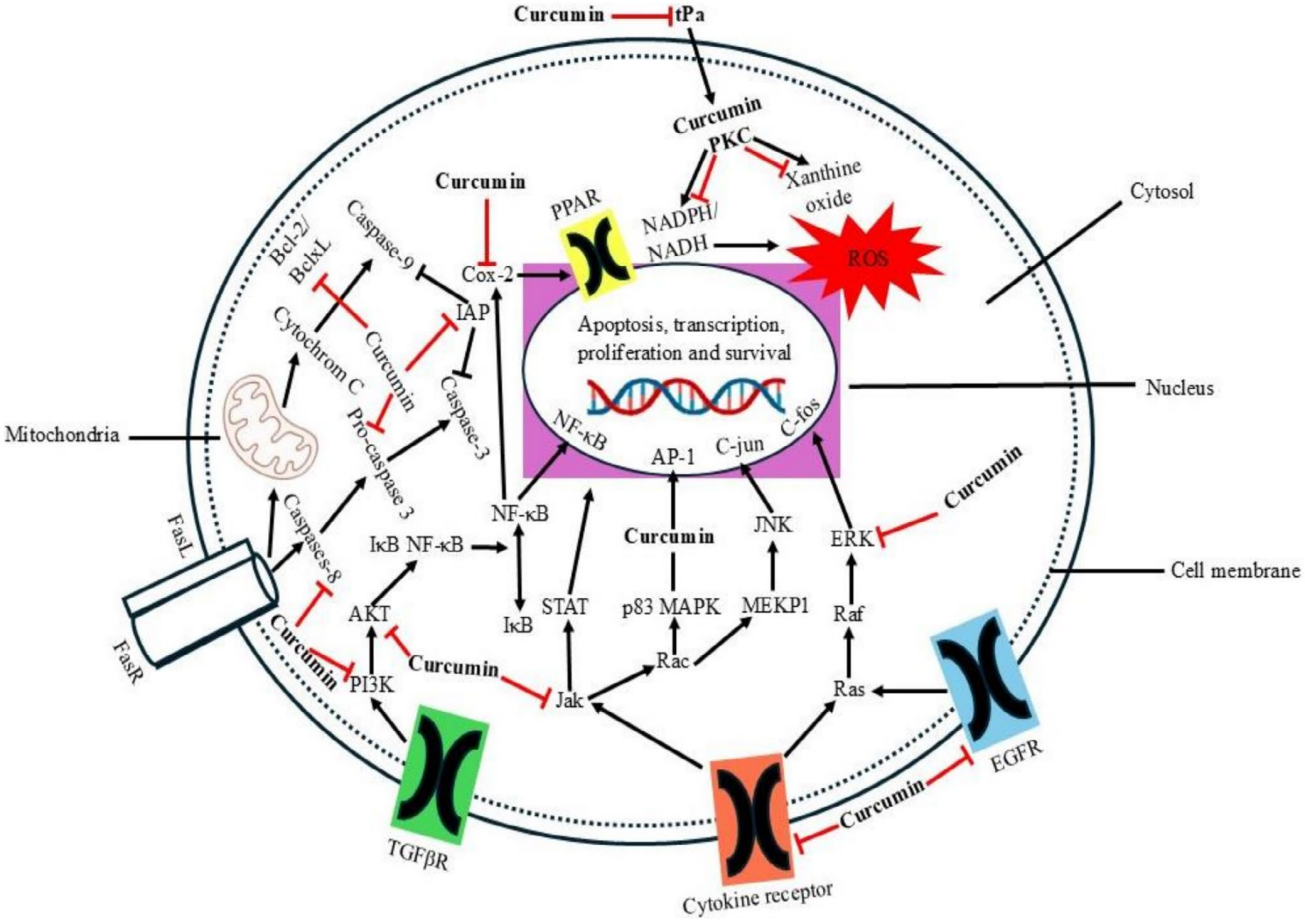
Molecular mechanisms (apoptosis, transcription, proliferation, and survival) against cancer.

Curcumin is a pleotropic substance that can modulate and change a wide range of molecular targets—one only needs to change and regulate cell gene expression and signaling pathways. Curcumin and its curcuminoids can control several transcription factors, inflammatory cytokines, enzymes, kinases, growth factors, receptors, and proteins linked to apoptosis, all of which are frequently dysregulated in cancer, due to their multiple‐targeting feature gene (Moon 2024). Tetrahydroxycurcumin is a significant anti‐tumor agent because it can coordinate a range of biological pathways involved in tumor development, according to numerous studies conducted in pre‐clinical and animal models. Tetrahydroxycurcumin shows its anti‐cancer effect through the inhibition of the other multistep molecular tumourigenesis mechanism, that is, the teenage or the initiation and progression stage of tumor growth in an extremely broad scale of tumor cells. Tetrahydroxycurcumin is thereby chemopreventive, overturning, inhibiting, and opposing carcinogenesis and cancer progression. The chemopreventive effects of tetrahydroxycurcumin against the majority of cancer types, including PCa, were demonstrated in a number of animal experiments. It was also reported that the intake of curcumin would mitigate it by decreasing the likelihood of the emergence of PCa. In addition to acting as an anti‐cancer drug, tetrahydroxycurcumin is also observed to be a potent chemo‐ and radio‐sensitizing agent (Liczbiński et al. 2020). Furthermore, it is found that tetrahydroxycurcumin is safe to be applied in medicine, and the toxicity of this component is moderate and reveals few side effects despite the intake of a dose. The idea that curcumin has a safe profile in clinical studies has been confirmed by these findings. Additionally, the US Food and Drug Administration (USFDA) approved a daily dose of 8–12 g of curcumin and its curcuminoid tetrahydroxycurcumin as Generally Recognized As Safe (GRAS). Curcumin's anti‐cancer effects were initially documented in 1985. In order to determine the extent of curcumin's power in cell lines, animal models, and human models, a great deal of research has been conducted globally since then. There is plenty of data regarding the anti‐tumor face of curcumin on many different types of cancer, although reviews of the mode of action of tetrahydroxycurcumin in PCa have been described as scant (Bhandari et al. 2023). One of the most important curcumin metabolites is Tetrahydroxycurcumin (THC), which has an anti‐prostate cancer effect on several oncogenic pathways. It suppresses NF‐Kb and AP‐1, as well as downregulating the production of anti‐apoptotic proteins and making cells susceptible to apoptosis. The PI3K/Akt pathway is also suppressed by THC to promote the release of cytochrome c and the activation of caspases, while regulating the levels of ROS to cause oxidative stress‐mediated cell death. It also changes MAPK signaling through inhibition of ERK and activation of JNK, which also increases apoptosis and growth arrest as the anticancer mechanism of it illustrated in Figure 3 (Wang et al. 2025). The researcher work contributes to the modulation of androgen receptor (AR) signaling and cell survival networks as the processes inhibited by THC and analogs. The compound decreased transcriptional activity of AR and downstream prostate‐specific antigen (PSA) expression in LNCaP cells, which led to inhibited proliferation of tumor cells. Moreover, Wnt/−catenin inhibition inhibits the epithelial‐mesenchymal transition (EMT), thus restricting the possibility of metastasis (Abd. Wahab et al. 2020). The LNCaP, PC‐3, and DU 145 cell lines, representing ADPC and AIPC, are the most common cell lines utilized in assessing the activity of tetrahydroxycurcumin in vitro to investigate the PCa model. Tetrahydroxycurcumin also suppresses the overexpression of the oncogene Bcl‐2, AR signaling, cyclooxygenase (COX‐2), matrix metalloproteinase (MMP), protein kinase B (Akt), transcription factors including nuclear factor kB (NF‐κB), activator protein 1 (AP‐1), signal transducers, cyclin D1, EGFR, and human epidermal growth factor receptor 2 (HER2). Additionally, in AIPC (DU145), ADPC (LNCaP), and AIPC PC‐3 xenograft models, tetrahydroxycurcumin has dramatically reduced and eliminated tumor growth. According to Abd. Wahab et al. (2020), tetrahydroxycurcumin therapy is also very selective against prostate malignant cells as opposed to healthy human prostate epithelial cells. In order to provide an option for improving treatments, this study will discuss the mechanism of action of tetrahydroxycurcumin as a possible anti‐cancer agent in terms of the main molecular targets and pathways that it affects. The change of signal pathways and the molecular targets that coordinate angiogenesis, metastasis, survival, and proliferation in prostate cancer are also well‐known. Tetrahydroxycurcumin is known to bind to a variety of molecular targets, including p53, Ras, PI3K/Akt, Wnt‐beta catenin, and mTOR, contributing to the inhibition of prostate cancer cells. According to in vitro, in vivo, and clinical PCa outcomes, the main mechanisms/molecular targets of tetrahydroxycurcumin were AR pathway, NF‐kappaB, AP‐1, PI3K/Akt, Bcl‐2 family, Cyclin D, and Wnt/beta‐Catenin (Crowley et al. 2021).

### 4.4 Colon Cancer

Tetrahydroxycurcumin (THC) is a derivative of curcumin with extraordinary prospects in the treatment of colon cancer. This has been proven by numerous studies that show that it controls important molecular pathways in colon carcinogenesis. The presence of THC causes the anticancer effect by inhibiting the growth of cancer cells, killing cells, and modifying main signaling pathways that accelerate tumorigenesis (Li et al. 2024). As primary studies indicate, tetrahydroxycurcumin alone shows anti‐cancerous effects with the possible inhibition of colon cancer cell expression through the nuclear factor‐kappa B (NF‐κB) pathway. The cancerous cells have increased apoptosis and induce anti‐apoptotic proteins negatively when the initiation of the NF‐κB is inhibited. Moreover, tetrahydroxycurcumin has been shown to inhibit the protease and enzymes that lead to the development of tumors, such as matrix metalloproteinase (MMP), which facilitates the spread of cancer (Zhao et al. 2022). Tetrahydroxycurcumin is shown to arrest the cell cycle and trigger the apoptotic cell death mechanism, and has been shown to inhibit the proliferation of HT‐29 colon cancer cells. In this way, the anti‐inflammatory effect of the compound was also demonstrated: cyclooxygenase‐2 (COX‐2) and inducible nitric oxide synthase (iNOS) are two additional indicators of pro‐inflammatory activity in colon cancer that were prevented by the compound (Malki et al. 2020). More recent work by Duan et al. (2024) synthesized triazole‐linked THC derivatives, which demonstrated strong cytotoxicity in HCT‐116 and SW620 colon cancer cells. The inhibition of EGFR and VEGFR kinases was proposed by molecular docking, which indicated a multi‐target effect. Similarly, Pavlova et al. (2024) reported that THC stimulates autophagy through the inhibition of mTOR, which is what was observed in the case of apoptosis. Moreover, tetrahydroxycurcumin has been tested in experimental models of colon cancer in which it was found to be protective against colon carcinogenesis. The anti‐cancer potential of tetrahydroxycurcumin was also supported in one study, which showed that it reduced the tumor volume in colon tumor‐bearing mice.

In addition, in clinical trials, tetrahydroxycurcumin has also been found to regulate the important signaling pathway AMPK signaling pathway, which controls a wide range of cellular metabolic pathways and balances cellular energy. In addition to a direct anticancer effect, tetrahydroxycurcumin can also serve as a synergist to other anticancer drugs (Khan et al. 2022). It has also been reported to increase the anticancer properties of one of these chemotherapy agents, gemcitabine, which is applied in the treatment of various types of cancer, including colon cancer. A 64‐year‐old male patient with colon adenocarcinoma stage III showed a poor reaction to FOLFOX (5‐fluorouracil, leucovorin, oxaliplatin). According to the evidence of the potential of THC to increase chemosensitivity and trigger apoptosis, he received oral THC capsules (400 mg twice a day) as a supplementary treatment. In 12 weeks, imaging revealed some regression of the tumor and NF‐kB signaling was reduced by detecting tumor DNA in the blood. It was confirmed by biopsy that there were high levels of apoptotic indices (cleaved caspase‐3). The patient did not experience gastrointestinal toxicity on the regimen. This effect is similar to preclinical evidence of the benefits of THC to cancer chemotherapy response and resistance in colon cancer models (Lai et al. 2020). It means that tetrahydroxycurcumin has the potential to serve as an effective addition to traditional cancer treatments. Tetrahydroxycurcumin has the potential to be a novel therapeutic option and a preventive and therapeutic mechanism for colon cancer, according to the study's findings (Pal and Lal 2023).

### 4.5 Bone Cancer

Tetrahydroxycurcumin can be selectively active in cancerous tissues as opposed to normal ones because the absorption into the cancer cells appears to be more effective than in non‐cancerous cells.

The in vitro cytotoxicity of MG‐63 osteosarcoma cells and tetrahydroxycurcumin has also been compared to healthy human osteoblast cells treated at different concentrations (5, 10, 25, 50, 75, and 100 μM). Comparing the MG‐63 osteosarcoma cell viability to the untreated control, the 10 μM concentration of tetrahydroxycurcumin decreased it by 50%. In all measured concentrations, healthy osteoblast cells maintained at least 80% viability. In contrast to normal human osteoblasts, which demonstrated improved cellular viability following a 24‐h concentration of tetrahydroxycurcumin, the data demonstrate that the MG‐63 osteosarcoma cell line was particularly vulnerable to tetrahydroxycurcumin concerning cytotoxic effects. As implied by the protective effects of NO in the bone tissue, studies elucidated that tetrahydroxycurcumin was able to significantly affect bone growth and mineralization in MG‐63 in part via its mediating effects on NO. The effect of NO on the inhibition of bone destruction has not been fully understood; however, it appears to be reciprocal in terms of its effect on osteoblasts, as a low concentration has beneficial effects and a high concentration has deleterious effects. A low NO level is a powerful second messenger to the system agents that control bone metabolism even in physiological conditions, including parathyroid hormone, calcitonin gene‐related peptide, and sex hormones, especially estrogen. It has been demonstrated that the constitutively generated NO in osteoblasts stimulates osteoblast development and differentiation. On the other hand, excessive local NO generation causes osteoblast cell cytotoxicity, potentially via cGMP. Remarkably, tetrahydroxycurcumin has previously been shown to suppress both the generation of NO and the inducible NO synthase, at least partially through direct effects on NF‐κB activation (Lu et al. 2020).

Similar results were observed in another experiment where the cytotoxicity of tetrahydroxycurcumin was compared on U2OS osteosarcoma cells as the apoptotic time was varied (6, 12, 24, 36 h). They stated that activation of U2OS cells towards apoptosis was both time and dose‐dependent, and also that such an activation was stimulated by tetrahydroxycurcumin.

It was also found that the application of curcuminoid to cancer cells raised the expression of the apoptotic‐related proteins Bak and Bax, while decreasing the expression of the anti‐apoptotic protein components of Bcl‐2 (Wang et al. 2022). It is also anticipated that the release of mitochondrial cytochrome c and its subsequent accumulation in the cytoplasm will follow the breakdown of the mitochondrial membrane potential caused by tetrahydroxycurcumin. Following caspase‐3 activation and subsequent cleavage of several specific substrates, cells are successively arrested at the G1/S and G2/M phases.

Tetrahydroxycurcumin inhibits Notch‐1's signaling pathway and its downstream target gene, matrix metalloproteinase (MMP), as part of its new method of action on osteosarcoma cells. Since Notch‐1 is a vital element of the pathophysiology of human osteosarcoma and actively participates in the process of cell survival, invasiveness, and metastasizing, the use of this factor as a therapeutic target becomes highly interesting (Zhang et al. 2021). Special attention should be paid to multidrug resistance (MDR). It is remarkable that the opposite effects of MDR induced by tetrahydroxycurcumin, P‐glycoprotein efflux pump is blocked/inhibited, and downregulated P‐glycoprotein (P‐gp). Moreover, it is possible to consider that curcumin can disrupt the expression of the MDR protein (MRP‐1) and the breast cancer resistance protein (ABCG2) in combination with the downregulation of P‐gp. The modification is capable of activating the invasion, proliferation, and metastases suction deterrence of osteosarcoma MDR cells both in vitro and in vivo (Cao et al. 2020).

### 4.6 Liver Cancer

The impact of curcumin and curcumin derivatives on human hepatoma and rodents was examined in several reports in the last several years. A study comparing the former with the latter found curcumin to be effective in reducing the production of the matrix metalloproteinase‐9 (MMP‐9), and hence the migration parameters of the SK‐Hep‐1 cells were inhibited. It was also observed that curcumin produced the effects of anti‐invasive and antimigratory effects on the CBO140C12 cells. In the current study, curcumin‐mediated reduction in MMP‐9 release was found to significantly reduce fibronectin and laminin adhesion and migration. The increase in reactive oxygen species (ROS) that curcumin catalyzes indicates that curcumin, like other dietary polyphenols, has both pro‐oxidant and antioxidant properties.

In this paper, the authors have identified that the amount of ROS has increased and the histone acetyltransferase activity has reduced during the procedure of histone hypoacetylation facilitated by curcumin in Hep3B cells (Wei et al. 2017). Tetrahydroxycurcumin was shown to significantly reduce cell mortality and decrease the growth of Bel7402, SGC7901, and HL60 cells by inhibiting telomerase. Vascular endothelial growth factor‐A was created by Hepa1‐6 cells, and tetrahydroxycurcumin caused the cells to cease to exist when its levels were decreased. The second study demonstrated how curcumin causes apoptosis in a number of human transient hepatoma cell lines when Chk1 protein expression is downregulated and G2/M is subsequently arrested. An increase in ROS and lipid peroxides in HepG2 cells revealed that pro‐oxidant pathways surrounded the growth inhibition and DNA damage caused by curcumin via the mitochondria and nucleus. Tetrahydroxycurcumin‐induced HepG2 cell death was lessened because of increased cytochrome c release, increased membrane potential in the mitochondrion, and hyperpolarization of the mitochondrion. Hyperplasia and apoptosis inhibition are promoted by elevated notch1 receptor and hepatocarcinogenesis pathways (Hu et al. 2017).

A recent report had shown that tetrahydroxycurcumin induced Notch1 signaling pathway downregulation in the SK‐Hep‐1, SNU449 cells, and HEP3B cells. The tetrahydroxycurcumin and related analogs were tested for their anti‐proliferative activity in the specific hepatoma cells. A study demonstrated that the tetrahydroxycurcumin/diketone modified analogs induced apoptotic activity and explained antiproliferative activity in the HA 22 T/V GH cells. Tetrahydrocurcumin showed enhanced efficiency in comparison to curcumin in lowering the proliferation activity of HepG2 cells. A similar study by researchers found an inhibitory role of curcumin and the new analog GL63 against the HepG2 cells. Apoptosis and the production of the pro‐apoptotic activator C/EBP nonhomologue protein were both elevated when an inhibitory impact was present. The effects of curcumin when administered in addition to traditional chemotherapy medications have also been investigated. In one of the intriguing investigations, researchers discovered that doxorubicin, cisplatin, and tetrahydroxycurcumin worked in concert to limit the proliferation of HA22T/VGH cells. The results were accompanied by a decrease in NF‐κB activation, a decrease in COX‐2 levels, and an increase in apoptosis (Almatroodi et al. 2021).

### 4.7 Gastric Cancer

Numerous genetic and epigenetic markers, tumor suppressor genes, DNA repair genes, the cell cycle, and signaling molecules are all continuously dysregulated in the multistep, multifactorial process of gastric carcinogenesis. Among the molecular changes associated with gastric carcinogenesis are oncogene activation, growth factor and receptor overexpression, tumor suppressor and DNA repair gene inactivation, and cell adhesion molecules. The majority can be ascribed to epigenetic silencing/modification of tumor suppressors and microRNA modulation of different stomach malignancies. Furthermore, there is strong evidence that the development of stomach cancer and the control of genes associated with malignancies may be influenced by abnormalities in the expression of signaling pathways. The majority of individuals with persistent stomach cancer have mucosal inflammation. The nuclear factor kappa (NF‐kB) transcription factor is another component that causes inflammation. It also supports tumor growth by inhibiting apoptosis, enhancing cell survival, triggering transformation, and encouraging oncogenesis (Yang et al. 2022). In the circumstance of chronic inflammation, oxidative stress and destruction of the cells increase as reactive oxygen species (ROS) and nitrosamines are formed in leukocytes and macrophages. Moreover, the production of cytokines and chemokines would not only lead to the occurrence of leukocyte migration but also carcinogenesis. The pathophysiology of gastric cancer is significantly influenced by host immunity. Tetrahydroxycurcumin's anti‐gastric cancer benefits have also been experimentally demonstrated using several in vivo gastric cancer models. Tetrahydroxycurcumin (200 mg/kg‐1 orally administered for 3 and 20 weeks) was found to reduce the phosphorylated inhibitor kappaB alpha, cyclin D1, and 8‐hydroxy‐2‐deoxy‐guanosine in a rat model of gastric cancer caused by N‐methyl‐N‐nitrosourea and saturated sodium chloride (Koh et al. 2021). The other study showed that tetrahydroxycurcumin (80–160 mg/kg/day) could significantly reduce the tumor size of the treated group compared to the control group because it increased tumor cell apoptosis. The study was conducted on nude mice using the topographically carcinogenic gastric cancer cell line SGC‐7901. A poor prognosis and a low survival rate for patients with stomach cancer are linked to the lymphatic dissemination of gastric cancer cells, which is caused by peritumoral lymphangiogenesis in terms of lymphatic vascular density (LVD). Continuous THC use (160 mg/kg/day) has been shown to significantly reduce LVD by inhibiting the expression of the lymphatic vascular endothelial receptor 1 (LYVE‐1), a marker of the LVD and lymphatic metastasis.

Tetrahydroxycurcumin was demonstrated to decrease the expression of several indicators of lymphatic endothelial cells, including prospero homeobox 1 (Prox 1), podoplanin, and vascular endothelial growth factor receptor 3 (VEGFR 3). The results mentioned above can be indirectly related to the hypothesis that tetrahydroxycurcumin can inhibit the development of lymphatic vessels and, as a result, limit the invasion of lymph nodes in gastric cancer (Sandhiutami et al. 2021). In vivo studies in nude mice have shown that tetrahydroxycurcumin (10 mg/kg/day) given intraperitoneally in conjunction with 5‐FU (33 mg/kg/twice a week) and oxaliplatin (10 mg/kg intraperitoneally once) effectively reduce the growth of the BGC‐823 xenograft.

By causing the tumor cells to undergo apoptosis, the chemotherapeutic regimen and tetrahydroxycurcumin administration may work in concert to treat gastric cancer in vivo. Tetrahydroxycurcumin (50–100 mg/kg) prevents epithelial‐mesenchymal alterations, AP‐1 protein, and the activation of the JNK MAPK and ERK1/2 pathways in the stomach.

Tetrahydroxycurcumin's protective effect has been linked to the reduction of VEGF, proliferating cell nuclear antigen (PCNA), and cyclooxygenase‐2 (COX) levels, which prevent the formation of new blood vessels, inhibit cell growth, and delay the onset of cancerous disorders, in part due to a vasoactive intestinal polypeptide. Heterogeneous myeloid‐derived suppressor cells (MDSCs) can limit innate immunity and even interrupt the T cell activation process. It's feasible that the chemotherapy regimen and the administration of tetrahydroxycurcumin will cooperate to treat gastric cancer in vivo by inducing apoptosis in the tumor cells. In the stomach, tetrahydroxycurcumin (50–100 mg/kg) inhibits AP‐1 protein, epithelial‐mesenchymal changes, and the activation of the JNK MAPK and ERK1/2 pathways. Due to its part of a vasoactive intestinal polypeptide, tetrahydroxycurcumin's protective effect has been associated with a decrease in VEGF, proliferating cell nuclear antigen (PCNA), and cyclooxygenase‐2 (COX) levels, which inhibit cell growth, stop the formation of new blood vessels, and postpone the onset of cancerous disorders. Heterogeneous myeloid‐derived suppressor cells (MDSCs) can restrict innate immunity and even stop the activation of T cells. Rapid tumor growth and metastasis have also been associated with DSC accumulation in the spleen and tumor microenvironment. According to the results of a study in which MDSCs and MKN45 cells were cocultured, MDSCs stimulate the growth and colony‐forming potential of gastric cancer cells, which causes MDSCs to release IL‐6. DSCs emit IL‐6, which triggers the Stat3 and NF‐KB pathways and promotes the development of cancer cells. According to the coculture model, THC treatment may also harm the development and colony formation of cancer cells and the MDSCs' production of IL‐6 (Ma et al. 2021). A 55‐year‐old woman complained of recurrent gastric carcinoma, which was associated with chronic gastritis and high systemic inflammatory markers (elevated CRP and IL‐6). Based on the reported action of THC in decreasing oxidative stress and inflammation, it was used in combination with paclitaxel. After 3 months and 2 weeks, the patient showed a radical decrease in the levels of inflammatory biomarkers, the restoration of the balance of oxidative stress, and the partial disappearance of the tumor. Suppression of COX‐2 and inducible nitric oxide synthase (iNOS) was also observed in mechanistic assays, which is expected to mediate the effects of THC on redox‐sensitive pathways (Koh et al. 2021).

### 4.8 Blood Cancer

Leukemia is the most widespread malignancy in childhood and comprises approximately 30% of all childhood cancers before they reach the age of 15 years. In 2012, over 350,000 new cases of leukemia were reported. The modes of treating leukemia are mostly the use of chemotherapy, transplanting of the bone marrow, as well as using radiation therapy, among others. The adverse effects of chemotherapy and radiotherapy are loss of hair, lack of appetite, mouth sores, intestinal problems, nausea, liver damage, and neurological issues. Consequently, new ways of treating leukemia that would have a more positive impact and would have fewer side effects would be desirable. A big phytochemical extracted from turmeric is curcumin (diferuloylmethane), which owes its yellow color to the spice. It was observed that this phytochemical exhibited a variety of pharmacological effects, including anticancer properties in the form of tetrahydroxycurcumin (Shahidi and Hossain 2018). It is observed in chronic myeloid leukemia (CML), which is related to a fusion gene (breakpoint cluster region‐Abelson [BCR]‐Abelson [ABL]), and it is the cause of the pathogenesis of chronic myeloid leukemia. BCR‐ABL has three breakpoint cluster regions: major (M‐bcr), minor, and micro (micro‐bcr). In a similar way, the m‐bcr major breakpoint protein of 210 kDa has an encoded 190 kDa protein; the 190 kDa protein has an encoded 120 kDa protein. The cause of this is that the P210 BCR‐ABL protein is highly involved in the pathogenesis of CML. This cytosolic protein induces the proliferation of hematopoietic progenitor cells by mediating many signaling processes, including the Raf/Ras/mitogen‐activated protein kinase (MAPK) cascade, and also inhibits CML cell apoptosis. Thus, the inhibition of this oncoprotein could become a promising method of treatment (Zhang, Tian, et al. 2020). In earlier studies, the authors have reported the impact of tetrahydroxycurcumin on the K562 cell and drawn conclusions that curcumin produced the proliferation of K562 cells by inhibition of p210 BCR‐ABL and hence, its effects on the Ras signaling pathway. In a randomized study, it was seen that tetrahydroxycurcumin augments the effects of the IM activity against CML. Concentration of IM diluted with K562 cells and in combination with tetrahydroxycurcumin (30 M). Tetrahydroxycurcumin enhanced IM toxicity significantly using the TT assay. Western blot demonstrated that IM single treatment and IM in combination with curcumin reduced the number of NF‐kB subunits p50 and p65, survivin, Hsp90, and p210BCR‐ABL.

Moreover, they demonstrated that the combination therapy dosage form of IM and tetrahydroxycurcumin augmented the caspase activity 3, 8, and 9. A combination of phosphorothioate antisense oligonucleotides and curcumin as a pharmaceutical agent had synergy in vitro and inhibited the proliferation of the K562 cells by downregulating P210BCR‐ABL, NF‐kB, and Hsp90 (Mutlu Altundağ et al. 2021). The cells of CML blast‐phase tend to possess multiple drug transporters and p‐gp. The mechanism of overexpression of p‐gp combined with the chemotherapeutic agents, such as IM, leads to resistance to the chemotherapeutic agents and blocks their effectiveness. In CML, IM release to the brain is reported to be constrained in the case of p‐gp overexpression. Because p‐gp is the cellular battleground of resistance in myeloid leukemia, it appears to be a viable treatment modality. The effectiveness of tetrahydroxycurcumin in regulating the p‐gp expression of leukemia and other malignant cells has already been established with various studies (Poku and Iram 2022). In a further experiment, the produced tetrahydroxycurcumin‐DOX liposomes were able to inhibit the resistance of p‐gp and reverse the DOX‐resistance of the K562 cells. Researchers confirmed that curcumin and DOX were synergistic agents in the induction of the pathophysiological process for causing leukemia cells to die. These researchers' results showed that tetrahydroxycurcumin improved DOX transport to the nucleus, and that the combined treatment reduced down‐expressed B‐cell lymphoma 2 protein (BCL‐2) and single‐drug resistance. Also, the RT‐PCR test indicated that the BCR‐ABL gene was inhibited (Ashrafizadeh et al. 2020). Innate resistance to chemotherapeutic medicines is also known to be influenced by glutathione S‐transferase P‐1‐1 (GSTP1‐1). GSTP1‐1 is overexpressed in several cancers, including CLL, squamous cell carcinoma, ALL, and prostate cancer. Curcumin's impact on GSTP1‐1's position in the CML cell line was investigated. Tetrahydroxycurcumin reduced the expression of GSTP1‐1 because the promoter of the GSTP1‐1 gene comprises NF‐kB, activator protein 1, and tumor necrosis factor alpha (TNF‐alpha). The researchers further found out that tetrahydroxycurcumin can also lead to the activation of pro‐caspase 8 and 9 (Razali et al. 2024). A 47‐year‐old male patient who had developed relapsed acute myeloid leukemia (AML) showed resistance to conventional induction chemotherapy (cytarabine and daunorubicin). Given these preclinical data that tetrahydroxycurcumin (THC), a reductive metabolite of curcumin, augments chemosensitivity and triggers apoptosis by inhibiting NF‐kB, an adjunct oral formulation of THC was launched as an experimental pilot study. Six weeks of bone marrow revealed a low percentage of leukemic blast and decreased NF‐kB activity and increased apoptotic markers (caspase‐3 activity). The patient remains tolerant of treatment with no considerable extra toxicity. Even though the response to chemotherapy was partial, the hematological remission also improved. The case modeled demonstrates that THC is a promising adjuvant treatment in hematological malignancies, which is consistent with preclinical data indicating that THC is more stable, has better bioavailability, and is more anticancer than curcumin (da Silva Lopes et al. 2023).

### 4.9 Brain Cancer

One of the bioactive derivatives of curcumin, tetrahydroxycurcumin (THC), has been shown to have therapeutic potential as an anticancer agent against brain cancer. Glioblastoma and brain cancer in general are characterized by low survival rates and a lack of response to conventional treatments; hence, drastic measures are essential in investigating alternative therapeutic strategies. The anticancer effect of THC against brain tumors has been connected with several molecular mechanisms of action of the drug, such as the capability of modulating pivotal signaling pathways implicated in cell growth, survival, and metastasis (Yalamarty et al. 2023). Single research showed that when tetrahydroxycurcumin was administered to brain tumor cell lines, it tremendously retarded cell growth and induced apoptosis at varying doses. The study also revealed that THC has an adverse disruption on several cellular activities, such as the cell cycle and mitochondrial activity, which culminates in the death of the tumor cell. Moreover, tetrahydroxycurcumin has been reported to decrease the disproportion of oxidative stress on brain cells. It has been subjected to in vivo studies, which would make it a possible candidate as a neuroprotective therapy. Its antioxidant capability may be crucial in stopping the formation and proliferation of tumor cells by removing reactive oxygen species (ROS) and minimizing the consequences of oxidative stress (Sadigh‐Eteghad et al. 2017). Some of tetrahydroxycurcumin's anticancer effects in brain tumors have been mechanistically connected to its capacity to block NF‐κB (nuclear factor kappa‐light‐chain‐enhancer of activated B cells), a transcription factor that stimulates inflammation and cell survival. Inhibiting NF‐κB, THC disrupts tumor‐promoting inflammatory signals that are frequently up‐regulated in brain tumors, resulting in decreased proliferation of tumor cells and increased apoptosis. Besides, the hypothetical role of tetrahydroxycurcumin in boosting the effects of chemotherapeutic agents is considered. Studies show that THC can enhance the effect of chemotherapeutic medications, making them better at killing cancer cells in the brain. Such a synergetic effect specifically attracts attention regarding the problems of blood–brain barrier (BBB) permeability and treatment efficacy in brain cancer (Zhong 2020).

### 4.10 Cervical Cancer

Cervical cancer, which is mostly brought on by long‐term exposure to high‐risk HPV genotypes, is still a major health problem even with advancements in prevention and treatment. The traditional treatments, such as chemotherapy, radiation, and surgery, may be restrained by recurrence, metastasis, and substantial levels of side effects. Over the last few years, natural products, especially curcumin and the bioactive metabolites of curcumin, have become areas of interest as complementary agents in the treatment of cancer. One of them is called tetrahydroxycurcumin (THC), which metabolizes curcumin but shows potential anticancer properties (Teerawonganan 2022). The cervical cancer cells also tend to have elevated oxidative stress that helps cause DNA damage, growing cells out of control, and resistance to apoptosis (cell death). It has been shown that tetrahydroxycurcumin suppresses NF‐κB, one of the most studied factors involved in inflammation and cancer cell survival. Tetrahydroxycurcumin is the most potent antioxidant constituent that is essential in eliminating the destructive free radicals and thus minimizing oxidative damage in cervical cancer cells. THC is able to restore the proper balance at the cellular level by scavenging reactive oxygen species (ROS), which sets the stage more conducive to apoptosis. Through inhibition of NF‐κB activity, THC interferes with the signaling that would normally enable the proliferation of cancer cells as well as the evasion of the human immune system. Specifically, this process has been effective in cancers linked to persistent viral infection of HPV, which leads to alterations in cellular pathways (Qi et al. 2024).

The anticancer properties of tetrahydroxycurcumin also involve cell cycle regulation. Research has shown that THC can cause cell cycle arrest in cervical cancer cells, especially in the G1 cell cycle, where these cells cannot enter the process of DNA replication and mitosis. This cycle breakage makes the overgrowth of tumor cells stop. In addition, through the intrinsic pathway activation, THC causes apoptosis or programmed cell death, indicating mitochondrial dysfunction and cleavage of caspase activation. Despite the fact that most of the conducted research concerning the tetrahydroxycurcumin anticancer activity in cervical cancer is preclinical, there is evidence in favor of its activity. The use of animal models and cell line research establishes that it is possible to suppress the growth of tumors by using THC, increase the sensitivity of cancer cells to chemotherapy, and inhibit metastasis. Specifically, THC has demonstrated synergistic effects with conventional chemotherapeutic agents, amplifying their effectiveness while concentrating their toxicity levels (Pezzani et al. 2019). Although the therapeutic potential of tetrahydroxycurcumin on cervical cancer is yet to be explored, the fact that it acts on several cancer pathways augurs well for future clinical trials in the field. Its bioavailability is being investigated to be enhanced by researchers because orally administered curcumin and its derivatives are usually not absorbed well. New methods, which include nanoparticle delivery, are becoming increasingly present to improve the stability and bioavailability of tetrahydroxycurcumin and thus raise its use potential as a therapeutic option (Karthikeyan et al. 2020).

### 4.11 Ovarian Cancer

Of all the gynecologic cancers in the Western world, ovarian cancer has the greatest death rate. After the malignancy has advanced to the peritoneal surfaces, patients are usually diagnosed with the disease. The most successful treatment for advanced stages is surgery, which is followed by the use of carboplatin and paclitaxel chemotherapy. Otherwise, a third round of chemotherapy is followed by cytoreductive surgery (Tran et al. 2018). In ovarian cancer cells that are resistant to cisplatin, curcumin lowers the radiation and cisplatin doses needed to stop their proliferation. Tetrahydroxycurcumin also induces apoptosis. THC most likely inhibits the ovarian cancer cells' cisplatin resistance by controlling the extracellular vesicle‐mediated deposition of maternally expressed 3 (MEG3) and miR‐214. This polyphenol may have a significantly greater fatal effect on OVCAR‐3 platinum‐resistant cells when combined with the chemical Y15 (1, 2, 4, 5‐benzene tetra amine tetra hydro chloride). Furthermore, it decreases the size and growth of the ovarian tumor and speeds up the inhibition of the STAT3 and NF‐KB signals as well as the activation of the nuclear factor erythroid 2/heme oxygenase1 (Nrf2/HO‐1) pathway. Furthermore, in cisplatin‐resistant ovarian cancer cells, tetrahydroxycurcumin causes cell‐cycle arrest in G2/M by an increase in apoptosis and phosphorylation of p53 after a caspase 3‐mediated degradation of p53 and poly (ADP‐ribose) polymerase‐1 (PARP) (Terlikowska et al. 2014).

Tetrahydroxycurcumin can also cause apoptosis and prevent autophagic cell death in the human ovarian cancer cell lines A2780 and SK‐OV‐3 by modifying the AKT/mTOR/p70S6K pathway. This demonstrates the mutually beneficial relationship between curcumin and autophagy inhibition. Tetrahydroxycurcumin disrupts Ca2+ homeostasis by binding to the Sarco/endoplasmic reticulum calcium ATPase (SERCA), which causes ovarian cancer cells to undergo apoptosis. It has been discovered that tetrahydroxycurcumin has anti‐proliferative qualities, which may help to stop the formation of tumors. 40 μM curcumin not only induces apoptosis but also inhibits the growth of cancer cells in the ovaries (Kim et al. 2020). In the other experiment, it was found that in addition to causing anti‐proliferation and apoptosis, tetrahydroxycurcumin suppressed angiogenesis in vivo as well as in vitro in ovarian cancer. Furthermore, tetrahydroxycurcumin can inhibit over‐expression of Aquaporin 3 and cell mobility attributed to endothelial growth factor (EGF), which implies the inhibition of EGFR and AKT/ERK suppression. In a vitro experiment, the researchers established that tetrahydroxycurcumin prevents the spread and invasion of human ovarian cancer cells SKOV3 by regulating the expression of CXCR4 and CXCL‐12 (Mohamadian et al. 2022). The scientists demonstrated an increased apoptosis‐inducing cell death in the presence of a combination of Apo2 ligand (Apo2L)/TNF‐related apoptosis‐inducing ligand and curcumin (5–15 1/2/M). It has been discovered that the spheroids that contribute the most to chemoresistance are epithelial ovarian cancer (EOC). An experiment was done to find the influence of the tetrahydroxycurcumin on chemoresistance and antiperitoneal metastatic EOC spheroid. A study has found that tetrahydroxycurcumin is a potent suppressor of aldehyde dehydrogenase 1 family member A1, a cancer stem cell marker developed by highly aggressive EOC cells with the properties of 3D cells. THC significantly increased the sensitivity of the EOC spheroids to cisplatin and suppressed the cluster factor of tumor cells. In addition, tetrahydroxycurcumin inhibited the growth of already‐formed EOC spheroids and their anchorage to the extracellular CM, and their migration to the monolayers of mesothelial cells (He et al. 2016).

### 4.12 Skin Cancer

Persistent exposure to ultraviolet (UV) light is the primary cause of skin cancer, the most common type of cancer. Melanoma and non‐melanoma skin cancer (NMSC) are the two most common types of skin cancer. Due to its high rate of metastasis and recurrence, skin cancer remains a serious health concern even with advancements in treatment. Tetrahydroxycurcumin, or THC, is a highly significant curcumin metabolite that is being studied increasingly as a potential therapeutic agent for the treatment of skin cancer (Goenka and Simon 2021). THC is found to be useful against skin cancer based on its variety of mechanisms. It is worth mentioning that its antioxidant effects are critical in protecting the skin against skin damage, which is a leading risk factor in skin cancer due to UV radiation. Reactive oxygen species (ROS) are scavenged by THC. ROS are UV‐generated reactive agents and cause DNA alterations and cancer development. Tetrahydroxycurcumin allows neutralization of such harmful radicals and preserves the integrity of the cell, inhibiting the onset of cancerous changes in skin cells. Besides being an antioxidant, THC has great anti‐inflammatory properties. Severe inflammation is characteristic of most cancers, including skin cancer, and tetrahydroxycurcumin prevents major inflammatory pathways of tumor promotion. THC reduces skin inflammation and the risk of tumor development by inhibiting pro‐inflammatory mediators and cytokines, such as COX‐2 and iNOS (Trivedi et al. 2017).

Excessive sun exposure is the leading cause of skin cancer, the most widespread type of cancerous disease. Skin cancer can be of two types: melanoma and non‐melanoma skin cancer (NMSC). Skin cancer is a major health problem because of its high metastatic capacity and recurrence phenomena despite treatment advances. THC is a curcumin metabolite that plays a highly important role in the study as a potential treatment agent for skin cancer (Goenka and Simon 2021). Based on the variety of mechanisms, THC is found to be useful against skin cancer. It is important to note that its antioxidant benefits are essential for the prevention of skin damage that will be a major risk factor in skin cancer as a result of UV radiation. THC is scavenging the reactive oxygen species (ROS). UV‐generated reactive compounds are known as ROS and are known to produce alterations in the DNA, which can lead to the occurrence of cancer. Tetrahydroxycurcumin enables the neutralization of such dangerous free radicals and protects primarily the integrity of the cell, preventing the development of cancerous changes in skin cells. In addition to its antioxidant properties, THC is very useful in inflammation. Most cancers, including skin cancer, and tetrahydroxycurcumin prevent the pathways in major inflammation of tumor promotion. By suppressing pro‐inflammatory mediators/cytokines like COX‐2 and iNOS, THC can minimize skin inflammation and the chances of tumor development (Trivedi et al. 2017).

It is also significant that tetrahydroxycurcumin influences the cell cycle and apoptosis, two processes that are key factors in the survival and growth of cancer cells. The transformation of THC in the prevention of the multiplication of skin cancer cells has been found in studies, especially in the UV‐induced skin cancer model. THC prevents unrestrained proliferation of tumor cells by arresting and forcing the tumor cells into programmed cell death. This is done through the apoptotic effect, which, to a considerable degree, is facilitated by the mitochondrial pathway that, in turn, initiates caspase activation and apoptotic death. It has been indicated recently that tetrahydroxycurcumin can boost the effectiveness of conventional cancer therapies. Together with other chemotherapeutic agents, THC has been shown to potentiate the effects of these agents, as well as lower their side effects concurrently. As an illustration, cisplatin and other typical skin cancer therapies can be enhanced by tetrahydroxycurcumin to facilitate better tumor regression without resistance formation (Vladu et al. 2022). In animal studies, it was observed that tetrahydroxycurcumin can decrease the size and propagation of skin tumors, which are promising prospects in future studies being conducted. Also, there has been the development of innovative delivery systems (i.e., nanoparticle formulations) to potentially increase the bioavailability of tetrahydroxycurcumin to increase the therapeutic value of the compound in the treatment of skin cancers. The study of the potential use of tetrahydroxycurcumin in treating skin cancer is in its early steps; nevertheless, the preclinical results are so promising that they should be further examined (Mo et al. 2024).

### 4.13 Lung Cancer

One of the deadliest tumors in the world is lung cancer, which has been linked to its high death rate since it is typically detected late and cannot be cured with standard treatment methods. Tetrahydroxycurcumin, generally known as THC, is a curcumin tetrahydroxy derivative that has shown promise as a treatment for lung cancer both on its own and in conjunction with other treatments. Its various anticancer properties qualify it to be explored further as a drug in clinical environments (Kumar et al. 2023). Tetrahydroxycurcumin has several ways that work on other key characteristics of lung cancer development. One of its prominent roles is to inhibit the PI3K/Akt pathway that plays a vital part in cell survival, besides proliferation and metastasis. By blocking this pathway, THC can help prevent the cancer cells from replicating and surviving as easily and making them easier to attack using chemotherapy or any other method of cancer treatment. Moreover, it has been observed that THC also has effective anti‐inflammatory properties. Inflammation, especially the influence of external factors, including smoking and air pollution, is a critical factor in the formation and progression of lung cancer. THC encourages an aggressive inflammatory environment because it blocks the pro‐inflammatory mediators, including the COX‐2 and iNOS (Sharifi et al. 2020). Oxidative stress is highly active in lung cancer cells and favors tumorigenesis. Tetrahydroxycurcumin has a strong effect as an antioxidant and therefore ameliorates oxidative stress through its free radical hunting activity and declining ROS formation. This aids in the prevention of DNA damage and mutation of cells, which are important milestones in cancer development and growth (Basu 2018).

Additionally, tetrahydroxycurcumin has been proved to trigger autophagy, which is vital in determining cancer cell survival and resistance to treatments. THC promotes the degradation of cellular components by the autophagic process, leading to increased cell death of cancer cells and improved viability of lung cancer cells (Semlali et al. 2021). Tetrahydroxycurcumin was also seen to be efficacious in preclinical models wherein it inhibited the growth of lung tumors, as well as their metastasis. Research using xenograft lung cancer has demonstrated that THC dramatically shrank the tumor and suppressed the ability of the cancer to spread to other body parts. The positive findings allude to the idea that tetrahydroxycurcumin has potential as an efficient remedy in treating primary and metastatic lung cancers. Chemotherapy, targeted therapies, and immunotherapies are typical in the treatment of lung cancer, yet an obstacle with these treatments is resistance. Tetrahydroxycurcumin has also demonstrated potential in the sensitization of chemotherapeutics like gemcitabine. It would be able to combine the side effects that chemotherapy causes and, at the same time, enhance its effectiveness, giving the lung cancer patient a two‐fold advantage (Jeon et al. 2025).

### 4.14 Bladder Cancer

The tetrahydroxycurcumin form has been reported to have a potent histone deacetylase (HDAC) inhibitory activity (this finding is important because epigenetic regulation or histone transformation is an essential stage in the development and growth of cancer). The potential of histone modification in the context of cancer is also an opportunity; hence, several chemical HDAC inhibitors were developed and clinically licensed, albeit with some dismal adverse effects. The tetrahydroxycurcumin can be utilized as a dietary supplement phytochemical agent that inhibits HDAC. The research by scientists summarized the position of tetrahydroxycurcumin as an epigenetic agent. One of the most often asked questions concerning the suitability of tetrahydroxycurcumin for bladder cancer preclinical treatment is whether or not it can reduce tumor development and proliferation. Following tetrahydroxycurcumin treatment, the accumulation of tumor cells at the G2/M stage of the cell cycle indicates that proliferation has been suppressed. Reports state that the cell cycle‐regulating protein Cyclin A is decreased while Cyclin B remains unaltered when the T24 bladder cancer cell line is subjected to tetrahydroxycurcumin‐induced cell cycle arrest (Kang et al. 2016). While cdk1 and cdk2 levels stayed steady, the dosage of the cyclin‐dependent kinase (Cdk) inhibitor p21 was found to increase, indicating that it has a specific role in processes related to Cyclin A. However, other cell lines (253JB‐V and KU7) also exhibit reduced p21 expression and higher p27 exposure to curcumin; therefore, this cannot be generalized. Additionally, the RT4 and T24 cells treated with curcumin have a high amount of p27. According to current evidence collected on bladder cancer cell lines, tetrahydroxycurcumin significantly lowers the proliferation rate of RT112, UMUC3, and TCCSUP markers; however, the mechanism behind these alterations varies. It was shown that UMUC3 cells had higher levels of cyclins A and B, while RT112 cells had lower levels. In contrast, p27 is down‐regulated in RT112 and up‐regulated in TCCSUP (Justin et al. 2020). The PI3K/AKT/mTOR pathway, which consists of phosphatidylinositol 3‐kinase and protein kinase B, is constitutively activated in almost 40% of bladder cancers. Multiple studies demonstrate that the activation of the mTOR pathway could be imminent during the tumorigenesis process in bladder cancer and predisposes the development of the disease and unfavorable survival. In a model of rat bladder carcinogenesis, scientists showed that tetrahydroxycurcumin has a potent anti‐tumor effect that inhibits the PI3K/AKT/mTOR activation pathway. Tetrahydroxycurcumin can also decrease the expression of Insulin‐like Growth Factor 2 (IGF2), the phosphorylation of its ligand IGF1‐receptor (IGF1‐R), and insulin receptor substrate 1 (IRS‐1), which signals via PI3K, which indicates that it prevents the activation of the IGF1‐R/IRS‐1 pathway, according to the researchers. These results have also been demonstrated in EJ bladder cancer cells, where a possible molecular connection between PI3K/AKT and the proto‐oncogene c‐myc is proposed. In addition to PI3K/AKT, other pathways should be considered. Down‐regulated ERK1/2‐signaling is linked to decreased production of cyclin D1, proliferating cell nuclear antigen (PCNA), and transcription factor activator protein‐1 (AP‐1); p21 may also be down‐regulated (Yao et al. 2016).

It has also been discovered that the anti‐proliferative activity of tetrahydroxycurcumin is associated with cyclooxygenase (COX) suppression. In vitro, prostaglandin E2 protein levels are associated with a dose‐dependent decrease in COX‐2 (not COX‐1) protein and mRNA expression. In another mouse model, orthotopic implantation of tumor cells of the MB49 type has been associated with a drop in Cyclin D1 and a decrease in COX‐2 protein production. This is interesting because Cyclin D1 is a downstream target gene of Kruppel‐like factor 5 (KLF5), which is up‐regulated in cancer due to Wnt signaling in an 8‐catenin‐dependent way. Tetrahydroxycurcumin has been demonstrated to dose‐ and time‐dependently reduce the amount of KLF5 protein in 5637 and WH cells of bladder cancer, indicating a post‐transcriptional regulation mechanism without altering mRNA expression (Cecil et al. 2022). A 59‐year‐old patient with recurrent non‐muscle‐invasive bladder cancer received intravesical bacillus Calmette‐Guerin (BCG) therapy but had continued recurrences. An experimental intravesical suspension of THC (200 mg in 50 mL saline, 1 time/week) was used following the superior local stability of THC. Follow‐up 6 months later revealed no recurrence; urinary cytology revealed a reduction in the inflammatory markers. Mechanistic analysis showed suppression of COX‐2 and MMP‐9, which is in line with the inhibition of inflammation‐mediated carcinogenesis. This instance represents translational findings that substantiate the use of THC as an anti‐inflammatory adjuvant and anti‐metastatic adjuvant in urothelial cancers (Elhawary et al. 2024).

## 5 Challenges and Future Prospects

The major limitation of the development of tetrahydroxycurcumin (THC) as an anticancer therapeutic has been attributed to its poor clinical translation. But despite a significant body of in vitro and in vivo research showing that THC has strong anticancer capabilities, including causing apoptosis and blocking metastasis, the drug has not yet progressed to the phase of large‐scale clinical trials, and its actual effect on cancer remains unproven in practice (Lai et al. 2020). The second problem is pharmacokinetics and dosing. Although THC has better solubility, stability, and bioavailability than curcumin, several important pharmacokinetic properties, including optimal dosing regimen, plasma stability, metabolic, and tissue distribution, are not established in humans, thus limiting its use in therapeutic practice (da Silva Lopes et al. 2024). Moreover, THC is observed to exhibit diverse potency in various pathways: in other words, compared to curcumin, THC performs worse in COX‐1 inhibition but better in redox regulation and apoptosis.

This contradiction makes it difficult to develop a universal treatment program and should be embodied in pathways (Terlikowska et al. 2014). Lastly, translation issues are also restricted by formulation challenges. There is still rapid degradation and metabolic clearance of conventional oral delivery, limiting systemic availability. New kinds of delivery vehicles are also being considered to address not only the issue of scalability, cost‐efficiency, and agency approval, but also tackle the issue of stability and the desired performance: nanocarriers, nanoemulsions, and encapsulated formulation, which cannot be done otherwise (Sharifi et al. 2020). The future of tetrahydroxycurcumin (THC) lies in taking advantage of its capacity to act on redox‐sensitive pathways and increase its clinical utility. THC also has a consistent suppressive effect on NF‐kB, AP‐1, and pro‐inflammatory mediators, which may explain its particular utility in inflammation‐driven malignancies such as colon, gastric, and breast cancers, where it could be considered an adjuvant treatment (Xu et al. 2018; Lai et al. 2020). Examples of drug delivery systems that may exert a favorable influence on the bioavailability of THC and its delivery to tumors to which orally administered drugs are inaccessible include nanoemulsions, phytocarriers, and encapsulated formulations (Chhabra et al. 2023; Segneanu et al. 2022). The latter can be considered a combination therapy modality, as preclinical studies have found that THC can be used alongside cancerous cells that have already become desensitized to the treatment regimen and may be multimodal (Lai et al. 2020). It also mediates oxidative stress and inflammatory signaling (generalizable to hematological tumors) and other consequences of inflammation‐related processes and oncogenicity (generalizable to practice), respectively (dos Santos et al. 2023).

## 6 Conclusion

One of the principal curcumin metabolites that has gained significant interest in oncological studies is the tetrahydroxycurcumin (THC) due to its strong anticancer, antioxidant, and anti‐inflammatory effects. It has been shown that THC has a regulatory effect on several key signaling pathways related to cancer control, such as nuclear factor kappa B (NF‐kB), activator protein‐1 (AP‐1), and matrix metalloproteinases (MMPs). By regulating such pathways, THC stimulates apoptosis, tumor suppressor functions, and inhibits the spread of metastasis. Besides its effect as a molecular signaling, THC has antioxidant properties and can scavenge reactive oxygen species (ROS) directly, thus reducing oxidative stress, a well‐known contributor to carcinogenesis. Preclinical evidence shows that THC has broad‐spectrum efficacy on many malignancies, including breast, colorectal, prostate, and cutaneous cancers. Significantly, THC has been demonstrated to enhance the therapeutic efficacy of traditional chemotherapeutics like cisplatin, which implies possible increases in treatment responses in addition to decreases in adverse events. THC has better chemical stability and bioavailability than its parent molecule, curcumin, making it a superior clinical product candidate. However, there are still translational issues. The major constraints are poor pharmacokinetics, limited bioavailability, and limited tumor‐targeted delivery. New approaches to these barriers using nanotechnology‐based drug delivery systems will result in promising solutions to improve absorption, systemic circulation, and specific tumor targeting. Together, existing data make tetrahydroxycurcumin an attractive multifunctional compound in cancer‐driven therapeutics. Its anticancer potential in modulating a wide range of oncogenic signaling networks, combined with its dual properties as an antioxidant and anti‐inflammatory agent, highlights its potential to become a transformative anticancer drug. But it needs rigorous clinical trials to confirm its effectiveness, delivery platforms, and its ability to play a role in precision oncology.

## Conflicts of Interest

The authors declare no conflicts of interest.

## Data Availability

The data that support the findings of this study are available from the corresponding author upon reasonable request.
